# Suprainguinal versus infrainguinal fascia iliaca compartment block for hip surgery: a systematic review and meta-analysis

**DOI:** 10.1097/MS9.0000000000005081

**Published:** 2026-05-21

**Authors:** Ahmed Ali A. Alshamrani, Ghassan Ali J. Alqahtani, Abdulaziz Aoudah T. Aldhabaan, Hassan Gharamah M. Alasmari, Mazin Abdulmohsin S. Sarhan, Mohammad Saad Alshomrani, Ahmed Khalofah M. Alahmari, Hassan Abdullah S. Alqahtani, Abdulaziz Abdullah M. Alghamdi, Shatha Adel A. Almasswary, Abdulrahman Saeed G. Alasmari, Abdulaziz Mesfer S. Alotaibi, Mohamed Al-Azab, Mohammed Shari Alshahrani

**Affiliations:** a Department of Anesthesia, Armed Forces Hospital South Region, Khamis Mushait, Saudi Arabia; b Department of Anesthesia, Aseer Central Hospital, Aseer Health Cluster, Abha, Saudi Arabia; c Department of Anesthesia, King Abdullah Hospital, Aseer Health Cluster, Bisha, Saudi Arabia; d Department of Anesthesia, Armed Forces Hospital, Al Hada, Taif, Saudi Arabia; e College of Medicine, King Khalid University, Abha, Saudi Arabia; f Department of Anesthesia, Prince Sultan Military Medical City, Riyadh, Saudi Arabia; g Faculty of Medicine, Sana’a University, Sana’a, Yemen

**Keywords:** fascia iliaca compartment block, hip surgery, infrainguinal, postoperative analgesia, suprainguinal

## Abstract

**Background:**

Effective postoperative analgesia is essential after hip surgery to facilitate early mobilization and reduce opioid consumption. The fascia iliaca compartment block (FICB) is commonly used for perioperative analgesia, with both suprainguinal and infrainguinal approaches described; however, their relative efficacy remains uncertain. This systematic review and meta-analysis compared the analgesic effectiveness of suprainguinal versus infrainguinal FICB in hip surgery.

**Methods:**

A systematic search of PubMed, Scopus, Web of Science, and Google Scholar was conducted from inception to the final search date. Randomized or prospective comparative studies involving adult patients undergoing hip surgery and directly comparing suprainguinal with infrainguinal FICB were included. Primary outcomes were time to first rescue analgesia and postoperative opioid consumption. Secondary outcomes included pain scores and adverse events. Data were pooled using random-effects models, and risk of bias was assessed using the Cochrane Risk of Bias 2 tool.

**Results:**

Seven studies comprising 660 patients met the inclusion criteria. Suprainguinal FICB significantly prolonged time to first rescue analgesia compared with the infrainguinal approach (mean difference 121.40 minutes; 95% CI 4.60–238.19), with substantial heterogeneity. Postoperative opioid consumption was significantly lower with suprainguinal FICB (standardized mean difference −1.35; 95% CI −2.29 to −0.41). Pain scores generally favored the suprainguinal approach, particularly between 6 and 24 hours postoperatively. No major block-related complications were reported, and adverse event rates were comparable.

**Conclusion:**

Suprainguinal FICB provides superior postoperative analgesia and reduces opioid requirements compared with the infrainguinal approach in patients undergoing hip surgery, supporting its preferential use within multimodal analgesia strategies.

## Background

Hip surgery, including hip fracture fixation and total hip arthroplasty, is frequently associated with severe postoperative pain that can adversely affect early mobilization, prolong hospital stay, delay functional recovery, and increase opioid consumption^[^1^]^. Inadequate pain control following these procedures has also been linked to higher rates of postoperative complications and reduced patient satisfaction^[^2^]^. As a result, effective postoperative analgesia is a cornerstone of perioperative care in hip surgery^[^3^]^.

HIGHLIGHTS

- Seven comparative studies (660 patients) evaluated suprainguinal vs infrainguinal fascia iliaca compartment block (FICB).
- Suprainguinal FICB significantly prolonged the time to first rescue analgesia.
- Postoperative opioid consumption was significantly lower with suprainguinal FICB.
- Pain scores favored the suprainguinal approach, especially 6–24 h postoperatively.
- Adverse event rates were similar, with no major block-related complications reported.

Contemporary pain management strategies increasingly emphasize multimodal analgesia, which combines different pharmacologic and regional techniques to target multiple pain pathways while minimizing opioid exposure^[^4^]^. Regional anesthesia techniques play a pivotal role in this approach by providing site-specific analgesia, improving functional recovery, and reducing opioid-related adverse effects such as nausea, vomiting, sedation, and respiratory depression^[^5^]^. Among these techniques, the fascia iliaca compartment block (FICB) has gained widespread acceptance for perioperative analgesia in hip surgery due to its relative simplicity, safety profile, and ability to provide broad sensory coverage of the hip and proximal femur^[^4^]^.

The FICB involves deposition of local anesthetic beneath the fascia iliaca to block the femoral nerve, lateral femoral cutaneous nerve, and, variably, the obturator nerve^[^6^]^. Traditionally, the block was performed using a landmark-based infrainguinal approach. With the advent of ultrasound guidance, the infrainguinal technique was refined, improving block accuracy and success rates^[^6^]^. However, inconsistent spread of local anesthetic to the obturator nerve and variable analgesic efficacy has been reported with this approach.

To address these limitations, a modified ultrasound-guided suprainguinal FICB technique was introduced^[^7^]^. By placing the injection site above the inguinal ligament, the suprainguinal approach facilitates cranial spread of local anesthetic within the iliac fossa, potentially resulting in more consistent blockade of the femoral and obturator nerves^[^7^]^. Several clinical studies have suggested that the suprainguinal approach may provide longer-lasting analgesia, improved pain control, and reduced postoperative opioid requirements compared with the infrainguinal technique^[^8^]^. Nevertheless, concerns remain regarding possible motor weakness due to femoral nerve involvement, which may affect early ambulation^[^9^]^.

Despite growing interest in the suprainguinal approach, existing evidence remains heterogeneous. Prior meta-analyses evaluating FICB in hip surgery have yielded conflicting results regarding analgesic efficacy, opioid consumption, and postoperative outcomes. Importantly, many of these analyses pooled data from landmark-guided, infrainguinal, and suprainguinal techniques, potentially obscuring clinically relevant differences between approaches^[^8^]^. Therefore, a focused comparison of suprainguinal versus infrainguinal FICB is warranted. This systematic review and meta-analysis aim to evaluate and compare the analgesic efficacy, opioid-sparing effects, and safety profiles of these two techniques in patients undergoing hip surgery, with the goal of clarifying their relative clinical value and informing evidence-based practice.

## Methods

### Study design

This study was conducted as a systematic review and meta-analysis to compare the analgesic efficacy of suprainguinal versus infrainguinal FICB in patients undergoing hip surgery. The review was designed and reported in accordance with the Preferred Reporting Items for Systematic Reviews and Meta-Analyses (PRISMA) 2020 guidelines and the Cochrane Handbook for Systematic Reviews of Interventions^[^10,11^]^. A predefined protocol guided the search strategy, study selection, data extraction, quality assessment, and statistical analysis. The study protocol was registered in the International Prospective Register of Systematic Reviews (PROSPERO; registration number CRD420261332895).

### Search strategy

A comprehensive literature search was conducted in PubMed, Scopus, and Web of Science from database inception to the date of the final search. The search strategy combined controlled vocabulary and free-text terms related to FICB and its anatomical approaches, including “fascia iliaca block,” “fascia iliaca compartment block,” “suprainguinal,” and “infrainguinal,” together with terms related to hip surgery such as “hip arthroplasty,” “hip fracture,” and “proximal femur.” Searches were limited to human studies published in English, with no restriction on publication year.

To ensure broader coverage, a supplementary search was performed using Google Scholar. Because Google Scholar ranks results according to relevance and citation metrics, the first four pages of search results were screened to maximize the likelihood of identifying eligible studies. This process yielded 40 records for screening. Google Scholar also indexes a wide range of academic materials, including articles, preprints, theses, and institutional reports, which may capture some gray literature sources. Reference lists of relevant articles and included studies were also manually screened to identify additional eligible publications. A formal search of dedicated gray literature databases (e.g., institutional repositories or preprint servers) was not performed. The detailed database-specific search strategies, including Boolean operators and exact search strings used for each database, are provided in Supplemental Digital Content File 1, available at: http://links.lww.com/MS9/B193.

### Inclusion and exclusion criteria

Studies were eligible for inclusion if they met the following criteria: (1) randomized or prospective comparative clinical studies; (2) adult patients undergoing hip surgery; (3) direct comparison between suprainguinal and infrainguinal FICB; and (4) reporting at least one relevant postoperative analgesic outcome, such as time to first rescue analgesia, opioid consumption, or pain scores. Studies that evaluated the suprainguinal FICB but did not include a direct infrainguinal comparator were considered eligible for qualitative synthesis if they involved a comparable hip surgery population and reported relevant postoperative analgesic outcomes. However, only studies directly comparing suprainguinal and infrainguinal FICB were included in the quantitative meta-analysis.

Studies were excluded if they were non-clinical (cadaveric, anatomical, or volunteer studies), review articles, editorials, case reports, or conference abstracts without full data. Studies involving non-hip surgical populations or mixed surgical cohorts without separable hip-specific data were also excluded. Articles that did not provide extractable outcome data or lacked a direct comparison between suprainguinal and infrainguinal approaches were excluded after full-text assessment.

### Study selection

All identified citations were imported into Rayyan for organization and duplicate removal^[^12^]^. Screening was conducted in two stages: an initial review of titles and abstracts, followed by a full-text assessment of potentially relevant articles. Four reviewers independently evaluated the articles according to the predefined eligibility criteria. Any discrepancies were resolved through team discussion or consultation with a senior reviewer. The study selection process is summarized in a PRISMA flow diagram. A list of full-text articles excluded after eligibility assessment, together with reasons for exclusion, is provided in Supplemental Digital Content File 2, available at: http://links.lww.com/MS9/B194.

### Data extraction

Data extraction was performed independently by multiple reviewers using a standardized data extraction form. Extracted data included study characteristics (author, year, country, and study design), patient demographics, type of hip surgery, details of the fascia iliaca block technique (approach, guidance method, local anesthetic type, and volume), and postoperative analgesic outcomes. Primary outcomes of interest were time to first rescue analgesia and postoperative opioid consumption, while secondary outcomes included pain scores and adverse events. Any disagreements during data extraction were resolved by consensus.

### Quality assessment

The methodological quality and risk of bias of included studies were assessed independently by reviewers using the Cochrane Risk of Bias 2 (RoB 2) tool for randomized trials. The tool evaluates bias across five domains: bias arising from the randomization process, bias due to deviations from intended interventions, bias due to missing outcome data, bias in the measurement of outcomes, and bias in the selection of the reported results. Each domain was judged as low risk of bias or some concerns, and an overall risk of bias judgment was assigned for each study. Any disagreements were resolved through discussion.

### Statistical analysis

All statistical analyses were conducted using R software (version 4.3.3) with the meta and metafor packages. Continuous outcomes were pooled using a random-effects model. Mean difference (MD) with 95% confidence intervals was calculated for time to first rescue analgesia, while standardized mean difference (SMD) was used for postoperative opioid consumption due to variability in opioid agents and dosing units across studies. Statistical heterogeneity was assessed using the *I*^2^ statistic, with higher values indicating greater heterogeneity. Sensitivity analyses using a leave-one-out approach were conducted for the primary outcome to evaluate the influence of individual studies on pooled estimates and to explore potential sources of heterogeneity.

Pain scores and adverse events were summarized narratively due to heterogeneity in outcome reporting and assessment time points. Assessment of publication bias was not performed because the number of included studies was below the minimum recommended threshold for reliable evaluation^[^13^]^. Funnel plot-based methods and statistical tests for asymmetry, such as Egger’s regression and Begg’s rank correlation, are known to have low statistical power and may produce misleading results when applied to fewer than 10 studies^[^13,14^]^. In accordance with established methodological recommendations, no formal assessment of publication bias was undertaken.

## Results

### Study selection

**Figure 1.**
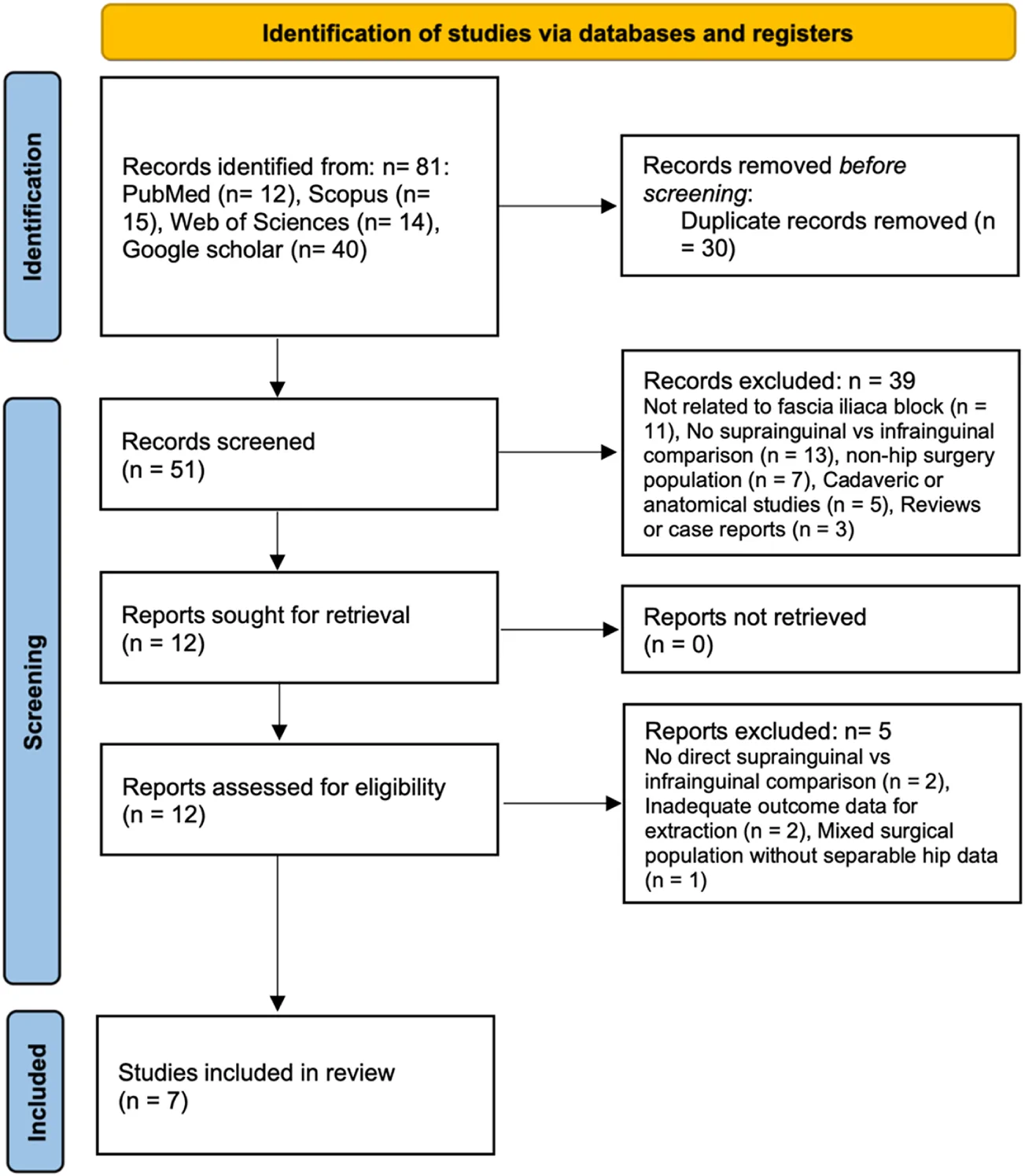
PRISMA 2020 flow diagram of study selection.

The literature search identified a total of 81 records from PubMed (*n* = 12), Scopus (*n* = 15), Web of Science (*n* = 14), and Google Scholar (*n* = 40). After the removal of 30 duplicate records, 51 records were screened based on title and abstract. Of these, 39 records were excluded for reasons including irrelevance to FICB, lack of suprainguinal versus infrainguinal comparison, non-hip surgical population, cadaveric or anatomical studies, and review articles or case reports. Twelve full-text reports were assessed for eligibility, of which five were excluded due to the absence of a direct comparison between suprainguinal and infrainguinal approaches, insufficient extractable outcome data, or mixed surgical populations without separable hip-specific data. Ultimately, seven studies met the inclusion criteria and were included in the systematic review and meta-analysis (Fig. 1)^[^7,15–20^]^.

### Study characteristics

The characteristics and methodological details of the included studies are summarized in Table 1. The seven studies were published between 2015 and 2025 and comprised a total of 660 patients. Sample sizes ranged from 32 to 200 participants. Six studies directly compared suprainguinal and infrainguinal FICB and were included in the quantitative meta-analysis. One study^[^16^]^ compared suprainguinal FICB with an anterior quadratus lumborum block rather than the infrainguinal approach and therefore did not meet the criteria for quantitative synthesis; however, it was included in the qualitative narrative synthesis because it evaluated the suprainguinal FICB technique in a comparable hip surgery population and reported relevant analgesic outcomes.

**Table 1:** Characteristics and methodology of included studies.

Study	Country	Study design	Sample size (total/groups)	Population/surgery type	Age (years, mean ± SD)	ASA class	Block technique (intervention)	Comparator	Guidance	Local anesthetic (type, conc., volume)	Anesthesia type
Kumar *et al*, 2015^[^15^]^	India	Prospective randomized controlled trial	40 (20 S/20 I)	Total hip arthroplasty	NR	ASA I–II	Suprainguinal FICB	Infrainguinal FICB	Landmark-based	Bupivacaine 0.2%, 40 ml	Spinal anesthesia
Bansal *et al*, 2022^[^7^]^	India	Prospective double-blind RCT	32 (16 S/16 I)	Above-knee lower limb surgeries (incl. hip)	~55	ASA I–III	Suprainguinal FICB	Infrainguinal FICB	Ultrasound-guided	Ropivacaine 0.2%, 30 ml	Spinal anesthesia
Refaat *et al*, 2023^[^16^]^	Egypt	Prospective randomized controlled trial	68 (34 FICB/34 QLB)	Hip surgery (femoral neck fracture)	40–60	ASA I–III	Suprainguinal FICB	Anterior QLB	Ultrasound-guided	Bupivacaine 0.25%, 40 ml	Spinal anesthesia
Lakshmi *et al*, 2024	India	Randomized controlled trial	200 (~100 S/ ~ 100 I)	Lower limb orthopedic surgeries	NR	NR	Suprainguinal FICB	Infrainguinal FICB	Ultrasound-guided	NR	NR
Jubairiya *et al*, 2024^[^18^]^	India	Prospective double-blind RCT	60 (30 S/30 I)	Proximal femoral fracture surgery	~74–77	ASA I–III	Suprainguinal FICB	Infrainguinal FICB	Ultrasound-guided	Ropivacaine 0.2%, 40 ml	Spinal anesthesia
Dwivedi *et al*, 2025^[^19^]^	India	Prospective randomized clinical trial	160 (80 S/80 I)	Elective hip surgeries	38–41	ASA I–II	Suprainguinal FICB	Infrainguinal FICB	Landmark-based	Bupivacaine 0.25%, 30 ml	Spinal anesthesia
Tata Niharika *et al*, 2025	India	Prospective randomized comparative study	100 (50 S/50 I)	Elective hip surgeries	~52	ASA I–IV	Suprainguinal FICB	Infrainguinal FICB	Ultrasound-guided	Ropivacaine 0.2%, 20–30 ml	Spinal anesthesia

FICB, fascia iliaca compartment block; S, suprainguinal; I, infrainguinal; QLB, quadratus lumborum block; NR, not reported.

Most studies were prospective randomized controlled trials, conducted predominantly in India, with one study from Egypt. All included studies involved patients undergoing hip surgery, including total hip arthroplasty, hip fracture surgery, or elective hip procedures. Fascia iliaca blocks were performed either using ultrasound guidance or landmark-based techniques. Local anesthetics commonly used were ropivacaine or bupivacaine at concentrations ranging from 0.2 to 0.25%, with injected volumes between 20 and 40 ml. All patients received spinal anesthesia for surgery.

### Time to first rescue analgesia

**Figure 2.**
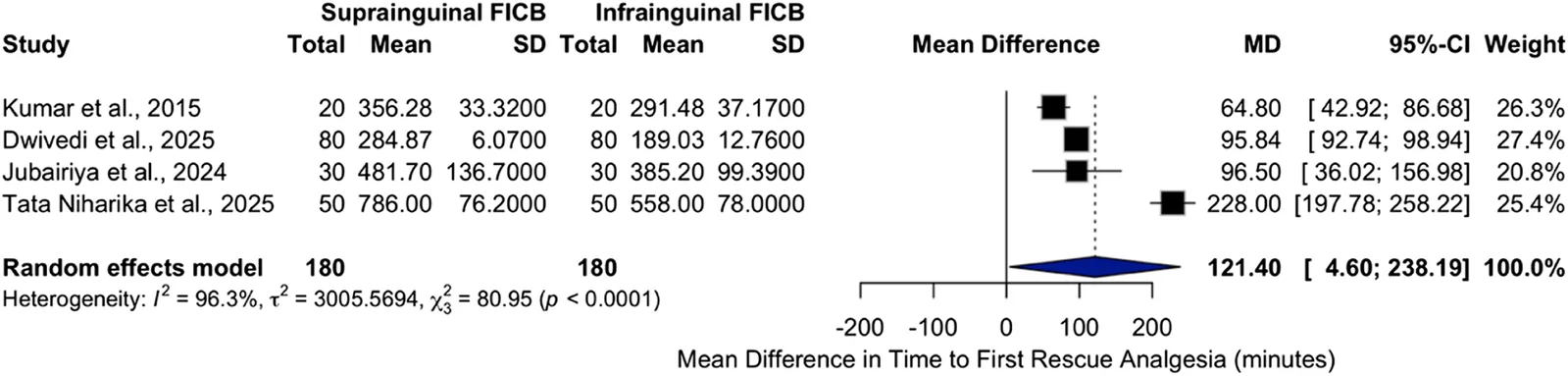
Forest plot comparing time to first rescue analgesia between suprainguinal and infrainguinal fascia iliaca compartment block.

Four studies reporting comparable data were included in the meta-analysis of time to first rescue analgesia (Fig. 2). Pooled analysis using a random-effects model demonstrated that suprainguinal FICB significantly prolonged the time to first rescue analgesia compared with the infrainguinal approach (MD 121.40 minutes; 95% CI 4.60–238.19 minutes). Substantial statistical heterogeneity was observed (*I*^2^ = 96.3%), likely reflecting variations in study populations, block techniques, and postoperative analgesic protocols. Individual studies consistently favored the suprainguinal approach, with longer analgesic duration reported across all included trials.

Results are expressed as mean difference (MD) in minutes with 95% confidence intervals using a random-effects model. Positive values favor the suprainguinal approach. Heterogeneity was assessed using the *I*^2^ statistic.

**Figure 3.**
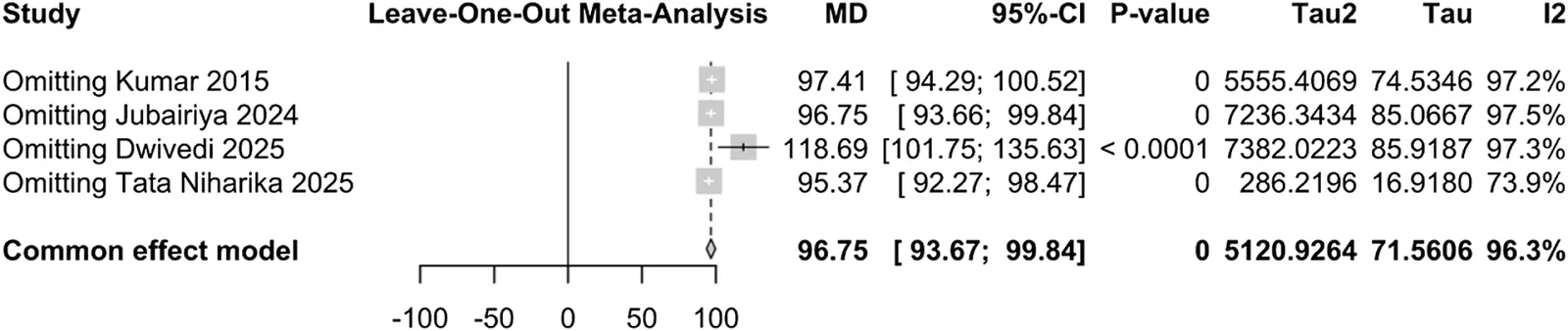
Leave-one-out sensitivity analysis for time to first rescue analgesia comparing suprainguinal versus infrainguinal fascia iliaca compartment block.

A leave-one-out sensitivity analysis was conducted to explore the robustness of the pooled estimate for time to first rescue analgesia (Fig. 3). Sequential exclusion of individual studies did not substantially alter the overall direction or statistical significance of the effect. The pooled MD remained consistently in favor of the suprainguinal approach, with effect estimates ranging from 95.37 to 118.69 minutes. The largest pooled effect was observed after removal of the study by Dwivedi *et al* (MD 118.69 minutes; 95% CI 101.75–135.63), whereas exclusion of Tata Niharika *et al* resulted in the smallest pooled estimate (MD 95.37 minutes; 95% CI 92.27–98.47). Although statistical heterogeneity remained high in most iterations, it decreased substantially after omission of the study by Tata Niharika *et al* (*I*^2^ = 73.9%), suggesting that differences in study methodology or patient population may partially contribute to the observed heterogeneity. Overall, the sensitivity analysis demonstrated that no single study disproportionately influenced the pooled effect estimate.

The figure shows the pooled mean difference (MD) in time to first rescue analgesia after sequential exclusion of each individual study. Squares represent pooled effect estimates after omission of a study, and horizontal lines indicate 95% confidence intervals.

### Postoperative opioid consumption

**Figure 4.**
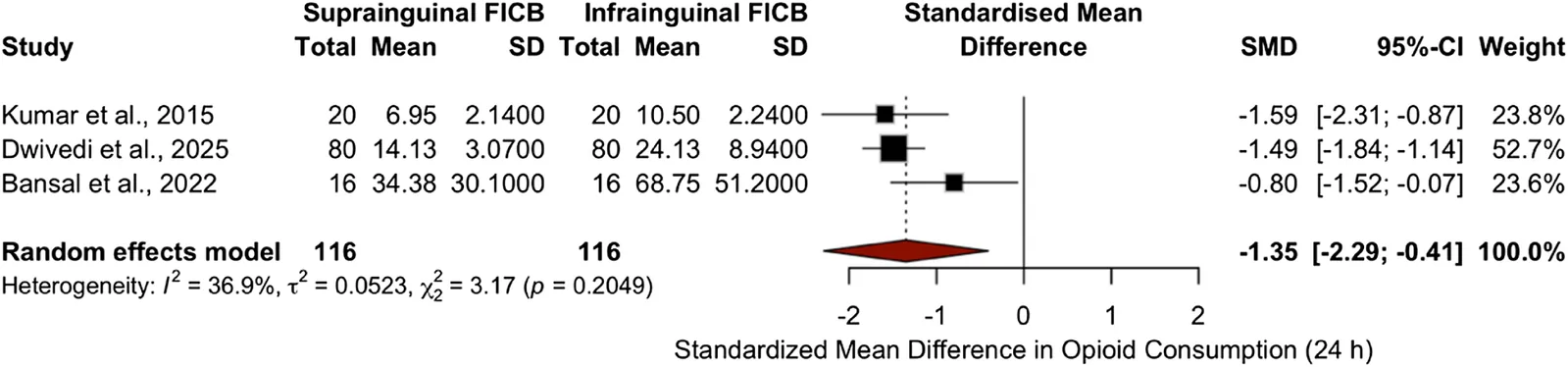
Forest plot comparing 24-hour postoperative opioid consumption between suprainguinal and infrainguinal fascia iliaca compartment block.

Three studies reporting 24-hour postoperative opioid consumption were included in the quantitative synthesis (Fig. 4). Due to differences in opioid agents and reporting units (morphine vs tramadol), results were pooled using the SMD. The meta-analysis demonstrated a significant reduction in postoperative opioid consumption in patients receiving suprainguinal FICB compared with infrainguinal FICB (SMD −1.35; 95% CI −2.29 to −0.41). Heterogeneity was moderate (*I*^2^ = 36.9%), indicating reasonable consistency among included studies.

Results are presented as standardized mean difference (SMD, Hedges’ *g*) with 95% confidence intervals using a random-effects model, due to differences in opioid agents and dosing units across studies. Negative values favor the suprainguinal approach.

### Pain scores

Pain outcomes were reported in all included studies; however, substantial heterogeneity existed regarding pain assessment tools [visual analog scale (VAS) or numeric rating scale (NRS)], time points, and conditions (rest vs movement). Consequently, pain outcomes were synthesized narratively (Table 2). Overall, suprainguinal FICB was associated with lower postoperative pain scores, particularly during the intermediate postoperative period (6–24 hours). Several studies reported significantly lower pain scores at specific time points, including at 6 hours, 12 hours, and 24 hours postoperatively, with some studies also demonstrating improved pain control during movement.

**Table 2:** Postoperative analgesic outcomes of included studies.

Study	Time to first rescue analgesia	Opioid/rescue analgesic consumption	Pain scores (VAS/NRS – key findings)	Adverse events	Main conclusion
Kumar et al, 2015^[^15^]^	**S:** 356 ± 33 min	24-h morphine:	Lower VAS at 6 h with suprainguinal; no difference at 12–24 h	Less nausea/vomiting in S group	Suprainguinal FICB provided superior analgesia and opioid sparing
	**I:** 291 ± 37 min	**S:** 6.95 ± 2.14 mg			
	*P* < 0.001	**I:** 10.50 ± 2.24 mg			
		*P* < 0.001			
Bansal et al, 2022^[^7^]^	No significant difference	24-h tramadol was significantly lower in S group (*P* = 0.028)	Lower NRS at 12 h and 20 h in S group	No difference in PONV	Suprainguinal FICB improved analgesia and patient satisfaction
Refaat et al, 2023^[^16^]^	**S:** 18 h (IQR)	24-h morphine:	NRS was similar overall; better comfort during spinal positioning in S group	No difference	Suprainguinal FICB superior to QLB for perioperative analgesia
	**QLB**: 2 h	**S:** 5.3 ± 0.9 mg			
	*P* = 0.005				
		**QLB**: 6.9 ± 1.87 mg			
		*P* = 0.009			
Lakshmi et al, 2024	NR	Lower opioid requirement in S group (*P* = 0.01)	Lower postoperative pain scores in S group	Similar complication rates	Suprainguinal FICB is more effective than infrainguinal approach
Jubairiya et al, 2024^[^18^]^	**S:** 482 ± 137 min	Paracetamol consumption similar	Lower VAS at 24 h in S group	No difference in delirium	Suprainguinal FICB provided longer analgesia
	**I:** 385 ± 99 min				
	*P* = 0.001				
Dwivedi et al, 2025^[^19^]^	**S:** 285 ± 6 min	Tramadol doses (24 h):	Lower VAS at 4, 6, 18, and 24 h in S group	Less hypotension, nausea, and vomiting in S group	Suprainguinal FICB is superior in analgesia and safety
	**I:** 189 ± 13 min	**S:** 14.1 ± 3.1			
		**I:** 24.1 ± 8.9			
	*P* = 0.008	*P* = 0.01			
Tata Niharika et al, 2025	**S:** 13.1 ± 1.27 h	Rescue tramadol required ~4 h earlier in I group	Lower VAS at rest and movement during 12–36 h with S group	NR	Suprainguinal FICB superior for intermediate-phase analgesia
	**I:** 9.3 ± 1.30 h				
	*P* < 0.001				

Data are presented as mean ± SD unless otherwise stated. Time to first rescue analgesia is reported in minutes or hours as originally described by individual studies; values were converted to minutes for meta-analysis where required. Pain scores were assessed using either the VAS or NRS, with higher scores indicating greater pain. Opioid consumption includes different opioid agents (e.g., morphine or tramadol) and units (mg or number of doses) across studies; therefore, opioid use was pooled using SMD in the meta-analysis. Due to heterogeneity in pain score reporting time points and outcome definitions, pain outcomes were summarized narratively rather than quantitatively pooled. FICB, fascia iliaca compartment block; S, suprainguinal; I, infrainguinal; NR, not reported.

### Adverse events

Adverse events were variably reported across studies. Most studies found no significant differences in the incidence of complications between suprainguinal and infrainguinal approaches (Table 2). However, one large study reported lower rates of hypotension, nausea, and vomiting in the suprainguinal group. No serious block-related complications were reported in any study.

### Risk of bias assessment

**Figure 5.**
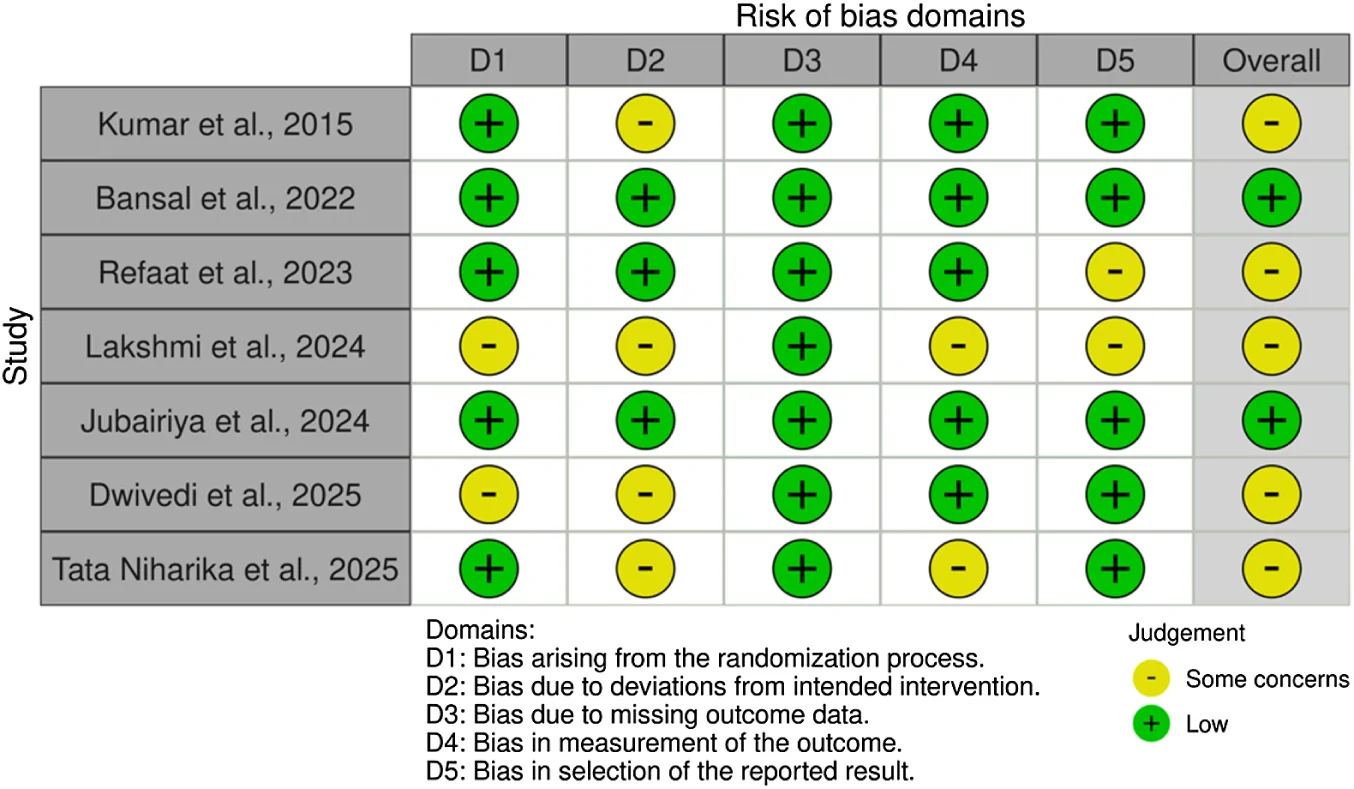
Risk of bias assessment of included studies using the Cochrane Risk of Bias 2 (RoB 2) tool.

Risk of bias was assessed for all included studies using the Cochrane RoB 2 tool, and the domain-level judgments are summarized in Figure 5. Overall, two studies^[^7,18^]^ were judged to be at low risk of bias, while the remaining five studies were assessed as having some concerns. Most studies demonstrated a low risk of bias in domains related to missing outcome data (D3) and measurement of outcomes (D4), reflecting complete follow-up and the use of objective or assessor-blinded outcome measures. However, some concerns were commonly identified in the domains of randomization process (D1) and deviations from intended interventions (D2), primarily due to incomplete reporting of allocation concealment and lack of blinding of block performers, which is typical in regional anesthesia trials. In addition, some concerns regarding selective reporting (D5) were noted in a few studies owing to the absence of pre-registered protocols.

## Discussion

This systematic review and meta-analysis demonstrate that the suprainguinal FICB provides superior postoperative analgesia compared with the infrainguinal approach in patients undergoing hip surgery. Across the included studies, the suprainguinal technique significantly prolonged the time to first rescue analgesia and reduced postoperative opioid consumption, with consistent direction of effect favoring the suprainguinal approach. Although heterogeneity was present, the findings across individual trials were largely concordant, supporting the robustness of the observed analgesic advantage.

The improved efficacy of the suprainguinal approach can be explained by its anatomical and pharmacological characteristics^[^21^]^. Injection above the inguinal ligament promotes cranial spread of local anesthetic within the iliac fossa, increasing the likelihood of consistent blockade of the femoral nerve and obturator nerve^[^21^]^. Given the substantial contribution of these nerves to the sensory innervation of the hip joint and surrounding structures, more reliable obturator nerve involvement may account for the longer duration of analgesia and reduced need for supplemental opioids observed in this analysis^[^22^]^. In contrast, infrainguinal injection is more prone to caudal and lateral spread, which may limit proximal nerve coverage and result in variable analgesic efficacy^[^15^]^. The leave-one-out sensitivity analysis confirmed the robustness of the primary finding that the suprainguinal approach prolongs postoperative analgesia compared with the infrainguinal technique. Sequential removal of individual studies did not materially change the pooled effect, indicating that the result was not driven by a single trial. Although heterogeneity remained substantial, it decreased after exclusion of the study by Tata Niharika *et al*, suggesting that differences in study characteristics may partly explain the observed variability.

Pain score outcomes were heterogeneous in terms of measurement tools and assessment time points; however, narrative synthesis revealed a tendency toward lower pain scores with the suprainguinal technique, particularly during the intermediate postoperative period^[^21^]^. This pattern is clinically relevant, as pain during the first 6–24 hours after hip surgery often interferes with early mobilization and rehabilitation^[^15^]^. The absence of consistent differences at very early or late time points likely reflects variations in perioperative analgesic protocols and the pharmacodynamic profile of local anesthetics rather than a lack of true analgesic benefit^[^21–23^]^.

Our findings help contextualize previously conflicting evidence regarding FICB in hip surgery. Earlier meta-analyses have reported mixed effects on opioid consumption, pain scores, and secondary outcomes, particularly in total hip arthroplasty and hip fracture populations^[^8^]^. A major contributor to this inconsistency is the inclusion of heterogeneous block techniques, including landmark-guided and infrainguinal approaches, which are known to provide less reliable obturator nerve blockade^[^24^]^. By directly comparing suprainguinal and infrainguinal approaches, the present study isolates the impact of technique refinement and suggests that earlier neutral findings may reflect dilution of treatment effects from less effective approaches.

The results of this analysis are consistent with individual randomized trials and prior syntheses that demonstrated reduced postoperative opioid requirements with suprainguinal FICB, particularly in total hip arthroplasty^[^25,26^]^. These findings align with earlier meta-analyses that reported opioid-sparing benefits of fascia iliaca block, while differing from more recent analyses that failed to demonstrate significant effects^[^26^]^. Differences in study selection, inclusion of non-randomized designs, pooling of surgical procedures, and failure to distinguish between block approaches likely explain these discrepancies.

A recent systematic review and meta-analysis by Yonghan Li evaluated the analgesic efficacy of suprainguinal FICB compared with control interventions in patients undergoing hip surgery^[^8^]^. In contrast, the present review focuses specifically on the technical comparison between suprainguinal and infrainguinal FICB approaches. By isolating studies that directly compare these two techniques, our analysis addresses a distinct clinical question and provides additional evidence regarding whether the suprainguinal approach offers advantages over the traditional infrainguinal technique. None of the included studies systematically evaluated quadriceps motor weakness or early ambulation outcomes, which limits conclusions regarding potential motor impairment associated with the suprainguinal approach.

Regional anesthesia continues to evolve in hip surgery, with increasing interest in motor-sparing techniques such as the pericapsular nerve group block^[^27^]^. While several meta-analyses suggest potential advantages of this approach over the traditional fascia iliaca block, direct comparisons with the suprainguinal technique remain limited and inconclusive^[^8,13,26^]^. Given the demonstrated analgesic efficacy of the suprainguinal approach in this study, future research should focus on head-to-head comparisons between modern ultrasound-guided techniques to better define optimal regional analgesia strategies. Taken together, the findings of this study support the suprainguinal FICB as a more effective regional analgesic technique than the infrainguinal approach for hip surgery. By reducing opioid requirements and prolonging analgesia, the suprainguinal technique may contribute meaningfully to enhanced recovery pathways and improved postoperative outcomes in this high-risk patient population.

Interpretation of the pooled results should consider the methodological quality of the included studies. Although two trials were assessed as having low risk of bias, the remaining studies were judged to have some concerns, primarily related to incomplete reporting of allocation concealment and lack of blinding of block performers. These limitations are common in regional anesthesia trials due to the practical challenges of blinding procedural interventions^[^28^]^. Nevertheless, most studies demonstrated low risk of bias in domains related to outcome measurement and missing data, and the primary outcomes analyzed in this review, such as time to rescue analgesia and opioid consumption, are relatively objective measures. Consequently, although methodological limitations may influence effect estimates to some extent, the overall consistency in the direction of effect across studies supports the reliability of the findings.

## Limitations

Several limitations should be considered when interpreting the findings of this systematic review and meta-analysis. First, although only comparative clinical studies were included, the overall number of eligible trials was limited, which may reduce the precision of pooled estimates and restrict the ability to perform detailed subgroup analyses. The relatively small number of studies also limited the feasibility of performing extensive subgroup or meta-regression analyses to fully explore sources of heterogeneity. In particular, variability in surgical indications, patient populations, and perioperative analgesic protocols may have contributed to the substantial heterogeneity observed for some outcomes. In addition, most included studies originated from a single geographic region (predominantly India), which may limit the generalizability of the findings to other healthcare settings and patient populations.

Second, differences in block techniques, including local anesthetic type, concentration, volume, and use of ultrasound guidance, may have influenced analgesic efficacy and limited the comparability of results across studies. In addition, variations in postoperative analgesic regimens and outcome assessment time points further complicated quantitative synthesis, particularly for pain scores and adverse events, which were therefore summarized narratively. Furthermore, several baseline characteristics and procedural details were incompletely reported in the primary studies (e.g., patient demographics, anesthetic dosing, or technique specifications), which may affect the precision of comparisons and interpretation of pooled outcomes.

Third, several studies had methodological limitations related to incomplete reporting of allocation concealment and blinding, which is common in regional anesthesia trials but may introduce performance or detection bias. Although the risk of bias was systematically assessed and most studies were judged to have low risk or some concerns, these factors should be taken into account when interpreting the results. Additionally, although a predefined protocol guided the conduct of this review, prospective registration was not completed prior to the literature search, which represents a methodological limitation when evaluated against current reporting standards. Finally, the relatively small number of included studies precluded a formal assessment of publication bias. While current methodological guidance discourages such analyses in small meta-analyses, the possibility of unpublished negative studies cannot be excluded. Although Google Scholar screening may capture some gray literature sources, dedicated searches of gray literature databases or trial registries were not systematically performed, which may increase the risk of publication bias or missed studies.

## Conclusion

This systematic review and meta-analysis suggest that the suprainguinal FICB appears to provide improved postoperative analgesia compared with the infrainguinal approach in patients undergoing hip surgery. The suprainguinal technique was associated with a longer duration of analgesia and reduced postoperative opioid consumption, with a generally consistent direction of effect across included studies. Although pain score outcomes were heterogeneous and the number of available studies was limited, narrative synthesis indicated a tendency toward improved pain control with the suprainguinal approach during the early to intermediate postoperative period. These findings support the consideration of the suprainguinal FICB as a potentially advantageous regional anesthesia technique within multimodal analgesia strategies for hip surgery. Nevertheless, further well-designed randomized controlled trials with standardized block protocols and outcome measures are needed to confirm these findings and to better define optimal regional analgesia strategies in this patient population.

## Data Availability

All data generated or analyzed during this study are included in this published article and its supplementary materials. Additional data are available from the corresponding author upon reasonable request.

## PROSPERO registration number

CRD420261332895.
